# Efficacy and Safety of a Novel Hemostatic Peptide Solution During Endoscopic Submucosal Dissection: A Multicenter Randomized Controlled Trial

**DOI:** 10.14309/ajg.0000000000002060

**Published:** 2022-11-30

**Authors:** Toshio Uraoka, Noriya Uedo, Tsuneo Oyama, Yutaka Saito, Naohisa Yahagi, Ai Fujimoto, Yoshiro Kawahara, Katsuhiro Mabe, Takuto Hikichi, Yorimasa Yamamoto, Hisao Tajiri

**Affiliations:** 1 Department of Gastroenterology and Hepatology, Gunma University Graduate School of Medicine, Maebashi, Japan;; 2 Department of Gastrointestinal Oncology, Osaka International Cancer Institute, Osaka, Japan;; 3 Department of Endoscopy, Saku Central Hospital Advanced Care Center, Nagano, Japan;; 4 Endoscopy Division, National Cancer Center Hospital, Tokyo, Japan;; 5 Division of Research and Development for Minimally Invasive Treatment, Cancer Center, Keio University School of Medicine, Tokyo, Japan;; 6 Department of Gastroenterology, National Hospital Organization Tokyo Medical Center, Tokyo, Japan;; 7 Department of Practical Gastrointestinal Endoscopy, Okayama University Graduate School of Medicine, Dentistry and Pharmaceutical Sciences, Okayama, Japan;; 8 Department of Gastroenterology, National Hospital Organization Hakodate National Hospital, Hakodate, Japan;; 9 Department of Endoscopy, Fukushima Medical University Hospital, Fukushima, Japan;; 10 Division of Gastroenterology Showa University Fujigaoka Hospital, Yokohama, Japan;; 11 Department of Innovative Interventional Endoscopy Research, The Jikei University School of Medicine, Tokyo, Japan.

## Abstract

To compare the effectiveness of the novel hemostatic peptide, TDM-621, with that of conventional hemostatic methods in treating intraoperative blood oozing during endoscopic submucosal dissection (ESD).

**METHODS:**

This multicenter, open-label, randomized controlled trial involved 227 patients with gastric and rectal epithelial tumors in whom ESD was indicated. Patients in whom the source of blood oozing was difficult to identify with waterjet washing during the procedure and required hemostasis with hemostatic forceps were randomly assigned to the TDM-621 and control groups. The TDM-621 group (in which hemostasis was achieved with TDM-621, followed by coagulation hemostasis with hemostatic forceps, as needed) was compared with the control group (in which hemostasis was achieved with hemostatic forceps). The primary end point was the mean number of coagulations with hemostatic forceps, determined by a blinded independent review committee. The secondary end points were the rate of achievement of hemostasis with only TDM-621, the dosage of TDM-621, and adverse events in the TDM-621 group.

**RESULTS:**

The mean number of coagulations with hemostatic forceps was significantly reduced in the TDM-621 group (1.0 ± 1.4) compared with that in the control group (4.9 ± 5.2) (*P* < 0.001). The rate of hemostasis achievement with only TDM-621 was 62.2%; the mean dosage of TDM-621 was 1.75 ± 2.14 mL. The rates of grade ≥3 adverse events were 6.2% and 5.0% in the TDM-621 and control groups, respectively.

**DISCUSSION:**

TDM-621 is a useful, easily operable hemostatic peptide for treatment of blood oozing during gastric and rectal ESD, with no serious safety concerns.

## Graphical abstract

**Figure:**
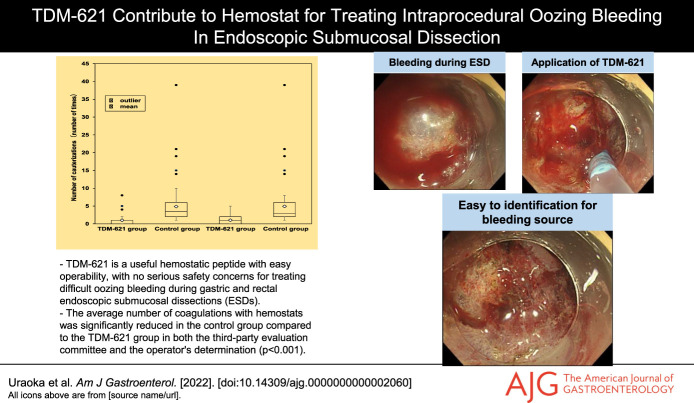

## INTRODUCTION

Great progress in technique and device development has been made in endoscopic resection of early-stage gastrointestinal cancer with endoscopic mucosal resection and endoscopic submucosal dissection (ESD) (1–3). However, the procedure time is extended, and an increased physical burden is placed on the patients and operators when complications, such as intraprocedural bleeding, occur. Although coagulation hemostasis is generally performed to manage intraprocedural bleeding during ESD (4,5), it is often difficult to identify the bleeding source before the application of hemostasis devices. In general, inaccurate coagulation hemostasis can increase the procedure time and risk of delayed perforation or localized peritonitis owing to excessive cautery burns (5–7). Although blood (fibrin glue, among others) and biological (collagen, among others) products are available for noninvasive hemostasis, especially in the surgical field, these products are not widely used in the field of gastrointestinal endoscopy because their endoscopic application is complex and they pose the potential risk of infectious viruses, prions, severe allergic reactions, and so on.

The synthetic self-assembling peptide, TDM-621 (3-D Matrix, Tokyo, Japan), is derived from nonliving sources and consists of 16 amino acid residues. It is usually in the form of an aqueous solution (that easily passes through the catheter) but can form a gel (self-assembling) under specific physiological conditions (neutral pH or an environment in which salts such as Na^+^ and K^+^ are present) (8). When applied *in vivo* during surgery, the bleeding site can be physically closed and bleeding can be stopped or decreased with the formation of a gel film on contact with blood in various tissues, including the brain and femoral artery, and the liver and skin of rats, hamsters, and mice (9). In animal experiments, this peptide solution has been shown to have several advantages, including improved wound healing (10,11).

TDM-621 has noninvasive properties, including safety and operability, and is a safe material that may contribute to hemostasis in the gastrointestinal endoscopic treatment field. However, no clinical studies have fully examined its efficacy and safety in the gastrointestinal field in a multicenter randomized setting. This multicenter, open-label, randomized controlled trial aimed to clarify the efficacy and safety of TDM-621 in achieving hemostasis during ESD in patients with gastric or rectal adenocarcinomas for which ESD is indicated.

## METHODS

This multicenter, randomized, open-label, parallel-group comparative trial was conducted from August 2017 to August 2019 with the approval of the Institutional Review Board of each facility in compliance with relevant laws and regulations, including the Declaration of Helsinki and the Japanese Ministerial Ordinance on Good Clinical Practice for Medical Devices. The study was registered in the University Hospital Medical Information Network clinical trial registration system (UMIN000042009). Before conducting the study, the principal investigator or subinvestigators explained the details of the study to the patients and obtained their written informed consent.

### Patients

Patients for whom ESD was indicated (following the Japanese gastric/colorectal cancer treatment guidelines and the gastric/colorectal ESD/endoscopic mucosal resection guidelines) (12–16), those who were aged 20 years or older during consent provision, and those with an epithelial tumor estimated to be >3 cm in the stomach (upper or middle third of the stomach, differentiated types of cancer that were preoperatively diagnosed as intramucosal cancer) or rectum (adenomas or intramucosal cancers and slightly submucosal invasive cancers) were included. Patients who were taking antithrombotic drugs were included only if the antiplatelet and/or anticoagulant drugs were discontinued before the procedure, according to the Japanese guidelines for gastroenterological endoscopy in patients undergoing antithrombotic treatment (16).

The exclusion criteria were as follows: residual or local recurrent lesions, ulceration of the target lesions, poorly differentiated adenocarcinoma or undifferentiated carcinoma of the stomach or rectum, inflammatory bowel disease, a history of hypersensitivity to peptide preparations or protein preparations, and a performance status grade of 2 or higher based on the clinical evaluation criteria for cancer chemotherapy. Patients who were considered ineligible for the study by a principal investigator or subinvestigators, those who were receiving blood coagulation drugs (hemocoagulase, among others) and antifibrinolytic agents (tranexamic acid and aprotinin preparations, among others), and cases in which oozing was not observed during surgery were excluded.

### Study protocol

ESD-trained endoscopists who had performed more than 20 ESD procedures participated in the study (17). To unify the procedure, all endoscopists registered 2 patients as roll-in cases regardless of the lesion site before the study commenced. A case review meeting was held once all the facilities had 2 roll-in cases, and the coordinating investigator decided whether to continue the study. Patients in 7 facilities who underwent ESD were randomly assigned in a 1:1 fashion to the TDM-621 and control groups by computer-generated dynamic allocation through an online system. The following measures were taken for patients in each group in whom identifying the bleeding source was difficult and hemostasis with coagulation forceps was required even after washing once with an endoscope water jet during ESD.

In the TDM-621 group, hemostasis was performed using TDM-621, and coagulation hemostasis was performed using hemostatic forceps (Coagrasper, Olympus, Tokyo, Japan) if hemostasis was not achieved within 3 minutes. TDM-621 was administered through a TDM-621 application catheter (FINE JET, Top, Tokyo, Japan) (see Supplementary Video 1, Supplementary Digital Content 6, http://links.lww.com/AJG/C782 and Supplemental Material 1, http://links.lww.com/AJG/C776, http://links.lww.com/AJG/C777, http://links.lww.com/AJG/C778, http://links.lww.com/AJG/C779, http://links.lww.com/AJG/C780). TDM-621 was applied to both the floor (gravity surface) and the ceiling (antigravity surface). In the control group, coagulation was performed using hemostatic forceps. To confirm the presence or absence of adverse events (AE), final observations were conducted 7 days after ESD. Follow-up examinations were performed in outpatient care 14 days after ESD. In addition, a telephone interview was conducted 28 days after ESD, and the presence or absence of AE was confirmed. The type of ESD knife or endoscope used by each practitioner was deemed irrelevant. Post-ESD closure was performed if the endoscopist deemed it necessary. A third-party evaluation committee—consisting of 3 highly experienced gastrointestinal endoscopists who were independent of the clinical trial organization—was established to evaluate videos of the procedures.

### Outcome measurements

The primary efficacy end point was the mean number of coagulations (performed with hemostatic forceps), as determined by the third-party evaluation committee.

The secondary efficacy end points were the mean number of coagulations (determined by the operator), time from confirmation of a blood oozing event to the completion of hemostasis (determined by the third-party evaluation committee and the operator), time from the initiation to the completion of ESD (determined by the operator), and the time from coagulation to the completion of ESD (determined by the operator).

Secondary end points for the TDM-621 group also included the number of sites in which hemostasis was achieved using TDM-621 alone, the number of sites in which bleeding recurred after achieving hemostasis, and the number of times those sites received additional treatment for hemostasis. The hemostasis and operation times were determined by the third-party evaluation committee and the operator. The amount of TDM-621 applied and the operability for the application of TDM-621 based on the operator's judgment were also evaluated in each case, based on 2 criteria: “it could be applied appropriately to the target site” and “it could not be applied appropriately to the target site.”

### Safety evaluation

The incidence of defects derived from TDM-621 treatment and AE (including abnormalities in clinical test values, postoperative bleeding for 7 and 28 days after ESD, and adverse reactions) were assessed for the evaluation of safety.

### Sample size and statistical analysis

The mean number of coagulations during ESD with hemostatic forceps (the primary efficacy end point) was assumed to be 3.1 in the control group and 2.1 in the TDM-621 group, based on our pilot study. Based on this assumed mean value, simulated data were created using random numbers according to a Poisson distribution. The required number of patients in each group, calculated with a simulation (1,000 times) to ensure equality of distribution in the 2 groups using a Wilcoxon rank sum test (detection power of 80% at the significance level), was 44. The SD of the total number of cauterizations was 2.3, while the SD in the Poisson distribution (mean value of 3.1) was 1.76. When the SD was 1.3, the required sample size was approximately 1.52; thus, the required sample size was adjusted to 80 patients in each group.

The primary, secondary, and other end points for efficacy were analyzed with the per protocol set (PPS) that represented compliance with the protocol. For the total number of cauterizations per patient treated with hemostatic forceps, a summary statistic (number of patients, mean, SD, median, minimum, maximum) was calculated. Superiority was confirmed with a significance level of 5% on both sides by comparing the groups using the Wilcoxon rank sum test. The number of cauterizations according to the operator's judgment was analyzed similarly to the primary efficacy end point. For other end points, summary statistics were calculated, and the Wilcoxon rank sum and Fisher exact tests were performed for intergroup comparisons. Safety was analyzed for the safety population (SP) and replaced as the lowest level term based on MedDRA/J version 22.1. Adverse reactions were classified with the system organ class and preferred terms and calculated using 95% confidence intervals. The Fisher exact test was used to compare the incidence rate between the groups, and statistical significance was set at *P* < 0.05. SAS software, version 9.4 (SAS Institute, Cary, NC), was used for statistical analysis.

## RESULTS

### Composition of patients

**Figure 1.**
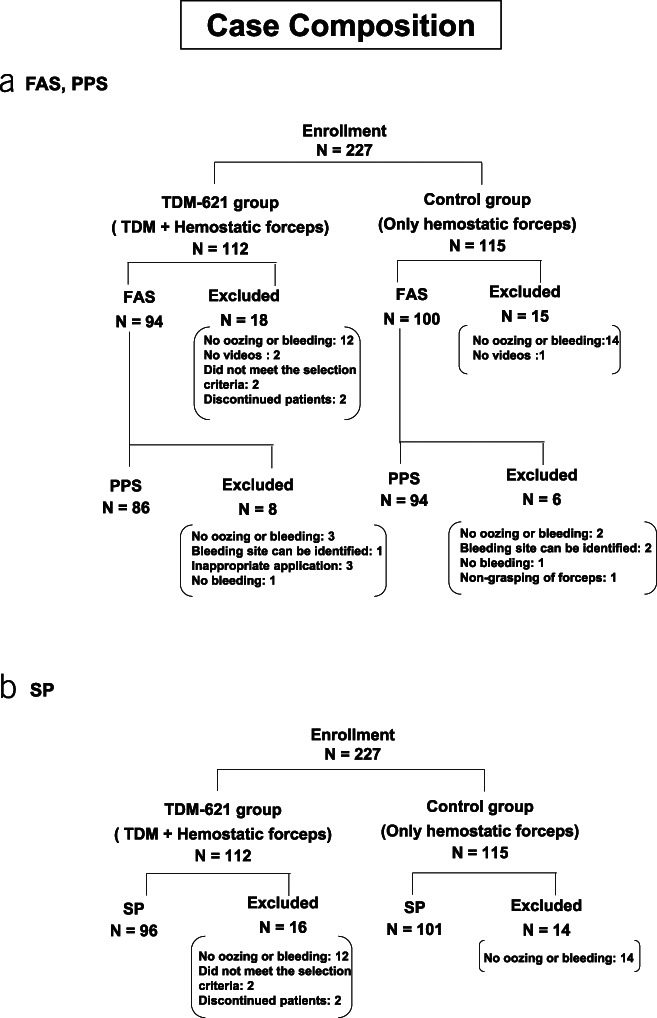
Composition of patients. (**a**) FAS and PPS. (**b**) SP. FAS, full analysis; PPS, per protocol sets; SP, safety population.

A total of 227 patients were enrolled from the 7 facilities and randomized (TDM-621 group, 112; control group, 115). Of them, 33 in both groups were excluded because of good clinical practice deviation, unavailability of record video, and misinclusion of bleeding in which bleeding source was identifiable after a single wash with a water jet during ESD (Figure 1a). The PPS, comprising 180 patients (TDM-621: 86, control: 94), excluded the following cases from the full analysis set, as determined by the third-party evaluation committee: deviation from the inclusion criteria, violation of exclusion criteria, noncompliance with hemostasis procedures, and improper use of research equipment. The SP included 197 patients (TDM-621: 96, control: 101) with blood oozing after a single wash using the water jet (Figure 1b).

### Demographic and other baseline characteristics

Table 1 summarizes the demographics and other baseline characteristics of the 180 patients included in the PPS. There were no remarkable differences in any demographic and baseline characteristic between the groups.

**Table 1.**
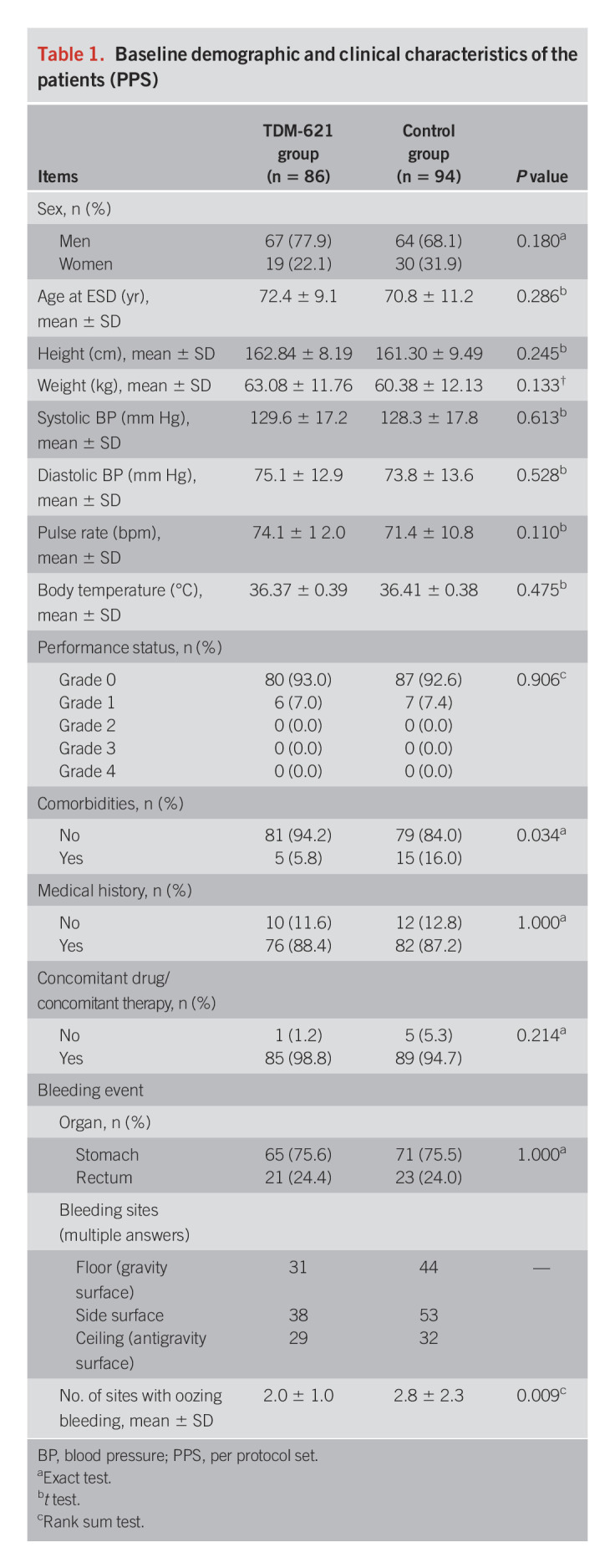
Baseline demographic and clinical characteristics of the patients (PPS)

### Primary and secondary efficacy end points

**Figure 2.**
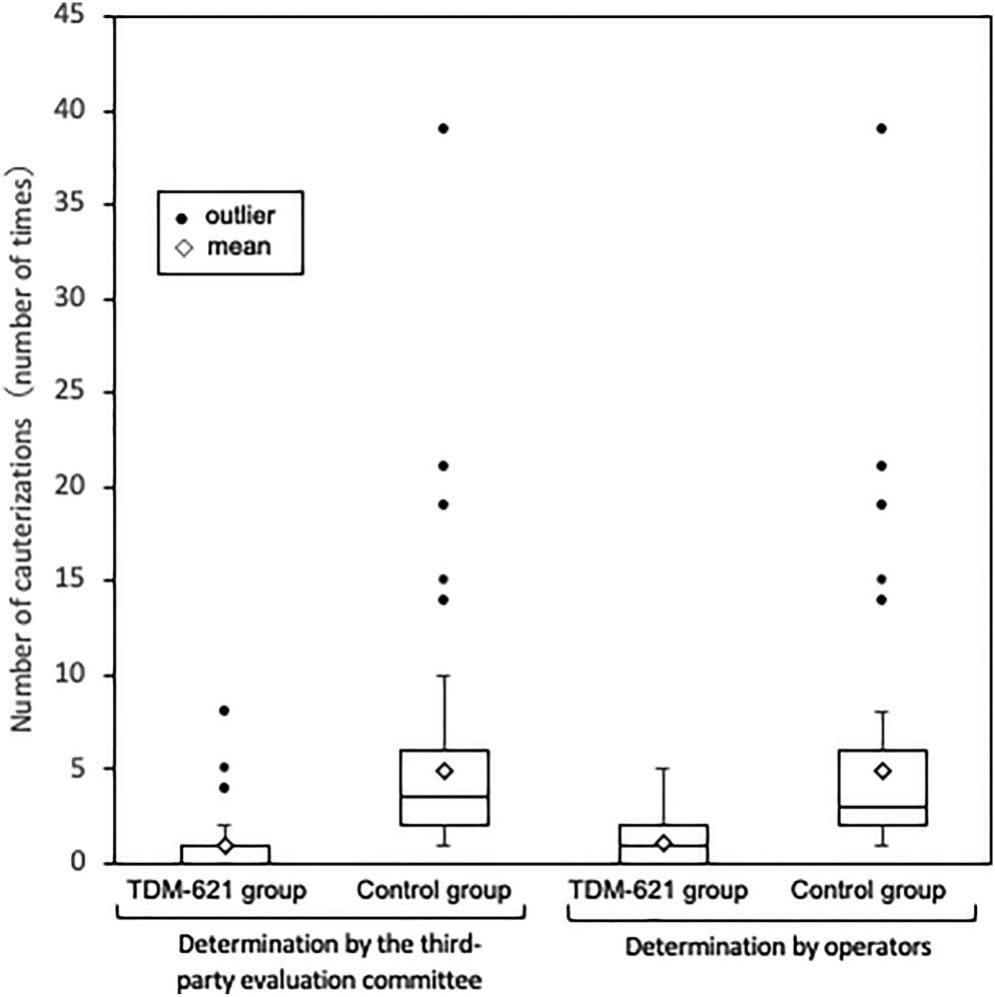
Effect of TDM-621 on the number of cauterizations with hemostats (PPS). Data are provided as mean ± SD. *P < 0.001 vs control group. BP, blood pressure; PPS, per protocol set.

The mean number of coagulations (the primary efficacy end point) determined by the third-party evaluation committee was significantly higher in the control group (4.9 ± 5.2) than in the TDM-621 group (1.0 ± 1.4) (*P* < 0.001) (Figure 2). Similarly, the number of coagulations determined by the operators (the secondary end point) was significantly higher in the control group (4.9 ± 5.3) than in the TDM-621 group (1.0 ± 1.4) (*P* < 0.001). There was no statistical difference between the evaluation by the third-party and that by the operator, nor between highly experienced (ESD experience ≥200) and less experienced (ESD experience <200) endoscopists (Supplement 2). Table 2 summarizes the other secondary efficacy end points. The time from the confirmation of blood oozing to the completion of hemostasis was significantly longer in the TDM-621 group than in the control group according to evaluation by the third-party evaluation committee and the operator (both *P* < 0.001). The time from the beginning to the completion of ESD (determined by the operator) was not significantly different between the TDM-621 and control groups. The proportion of patients without postcoagulation bleeding (determined by the operator) was 76 (88.4%) in the TDM-621 group and 86 (91.5%) in the control group, a nonsignificant difference (*P* = 0.620).

**Table 2.**
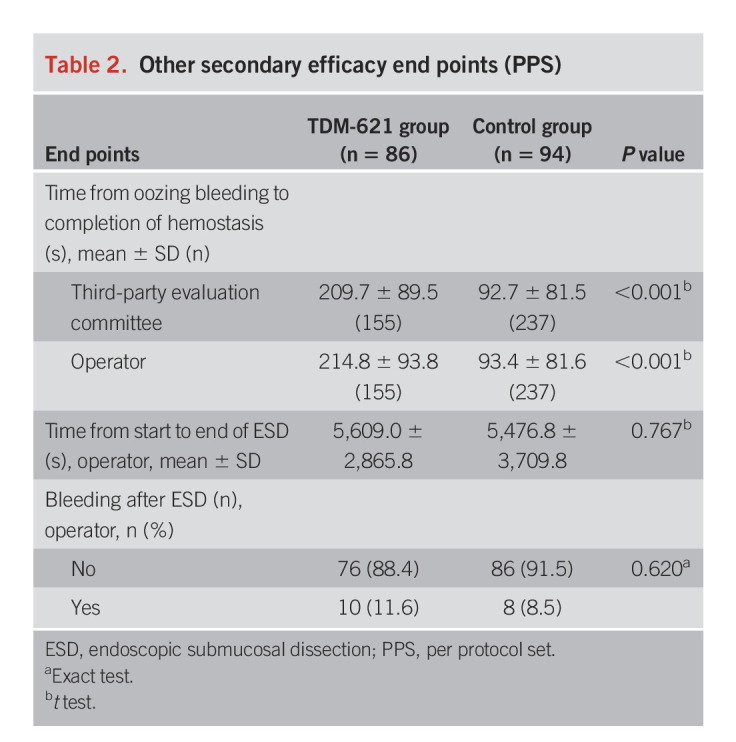
Other secondary efficacy end points (PPS)

Table 3 summarizes the hemostasis condition in the TDM-621 group. The hemostasis achievement rate with TDM-621 treatment alone was 61.4% ± 41.4%, as determined by the operator, and 62.2% ± 42.3%, as determined by the third-party evaluation committee, with no significant differences noted between the groups for both determinations. The error range of the TDM-621 single hemostasis achievement rate was large because the number of bleeding sites in each patient was small, the minimum value was 0%, the median value was 77.5%, and the maximum value was 100% (a rate of 100% indicates bleeding at 1 site, 66.6% indicates bleeding at 2 of 3 sites, etc). There was no recurrence of bleeding once hemostasis was achieved, and no additional treatments were required.

**Table 3.**
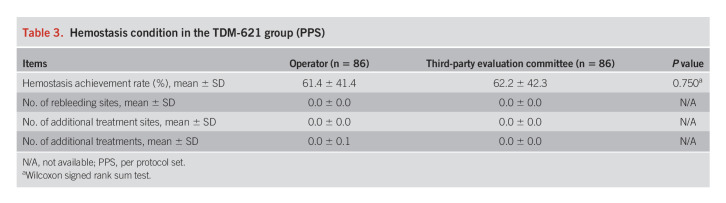
Hemostasis condition in the TDM-621 group (PPS)

### Other end points

The amount of TDM-621 applied was 1.75 ± 2.14 mL according to the operator's determination, and the operability was evaluated as “appropriately applied to the target site” in 85 patients (98.8%), all but 1. All targeted tumors were successfully removed *en bloc* in both groups.

### Safety results

The AE incidence rate was 39.6% in the TDM-621 group and 37.6% in the control group, with no significant differences between the groups. The incidence rate of adverse reactions was 1.0% in the TDM-621 group, and no adverse reactions were observed in the control group. No defects derived from TDM-621 occurred in any patients.

Table 4 summarizes the frequency of AE based on the Common Terminology Criteria for Adverse Events in the TDM-621, control, and roll-in groups. Post-ESD closure using endoclips was performed by operators' judgment in 2.3% (2/86) and 0% (0/94) of the TDM and control groups, respectively. The severity of these AE was mostly grade 1 or 2. Regarding serious AE, there were 2 patients (2.1%) with postoperative bleeding and 1 (1.0%) each with bacteremia, hematemesis, rectal perforation, and jaundice in the TDM-621 group and 3 patients (3.0%) with postoperative bleeding and 1 (1.0%) with gastric perforation in the control group. All patients recovered with conservative treatment. No AE requiring discontinuation of the study or leading to death occurred.

**Table 4.**
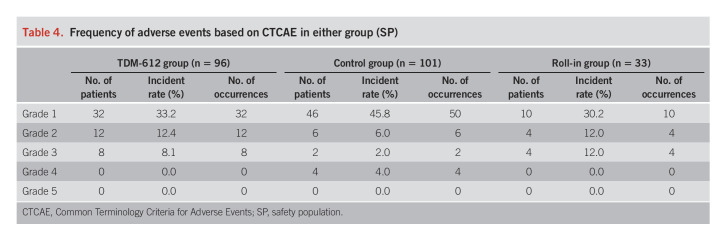
Frequency of adverse events based on CTCAE in either group (SP)

Regarding symptoms of post-ESD coagulation syndrome, such as fever, localized abdominal pain, and inflammation, several cases were observed in both the control and TDM-621 groups; however, there was no significant difference in the incidence between the 2 groups.

## DISCUSSION

This study showed that TDM-621 is a useful hemostatic peptide for blood oozing during ESD for gastric or rectal tumors and that severe AE do not occur with this peptide solution.

This is the first multicenter, randomized, parallel-group comparative study to directly compare the efficacy and safety of TDM-621 with those of conventional hemostatic methods for blood oozing during endoscopic resection. In this study, the number of coagulations with hemostatic forceps (the primary efficacy end point) was significantly reduced in the TDM-621 group compared with that in the control group. TDM-621 showed the efficacy to reduce the number of coagulations with hemostatic forceps by both highly and less experienced endoscopists.

A significant decrease in the number of cauterizations (the secondary efficacy end point) was shown, as determined by the operator, verifying the usefulness of TDM-621. The degree of dispersion of the number of cauterizations was markedly smaller in the TDM-621 group than in the control group. This result provides evidence that TDM-621 treatment reduced the number of coagulations considerably and suggests that treatment with TDM-621 can reduce the physical burden on patients during the procedure. There was no difference in the incidence of postcoagulation bleeding between the TDM-621 and control groups, which suggests that the hemostatic effects of TDM-621 were similar to those of the usual hemostasis methods during the procedure. The hemostasis achievement rate with TDM-621 treatment alone was 62.2%.

The time from blood oozing to achievement of hemostasis was significantly longer in the TDM-621 group than in the control group. This result may be because we set the time from the beginning of the TDM-621 treatment to the achievement of hemostasis to a maximum of 3 minutes. Furthermore, the time required for coagulation might increase while preparing the hemostatic forceps for coagulation in cases where hemostasis is not achieved. In clinical practice, the use of TDM-621 is expected to reduce the overall duration of ESD. For patients in the TDM-621 group in whom hemostasis was achieved, the operator's opinion was that the use of TDM-621 weakened the bleeding momentum and made it easier to identify the bleeding site. Consequently, TDM-621 might have contributed to the reduction in the number of coagulations even when hemostasis was not achieved.

TDM-621 has been available for use in preventing intraoperative ESD and post-ESD bleeding in the colon in 28 countries, including the United Kingdom, Germany, France, and Italy, since 2014, and was approved for use in Canada in 2019. In 2021, the US Food and Drug Administration approved this solution as a hemostatic agent. In Japan, TDM-621 has been found effective in achieving hemostasis in 25 patients who underwent cardiovascular surgery (18) and in 12 patients who underwent gastrointestinal surgery (19); however, those numbers were derived from single-arm, single-center pilot trials. Furthermore, in a single-center, single-blinded, randomized controlled trial (20) involving 91 patients with submucosal tumors and esophageal and colorectal deep infiltration lesions, the usage rate of an ESD knife tip or coagulation forceps for hemostasis was significantly reduced (49.3% vs 99.6%, *P* < 0.001) in the TDM-621 group compared with the group that did not receive TDM-621. These results indicate that TDM-621 is an efficacious, safe, and easy-to-use hemostatic peptide for the control of bleeding during endoscopic resection. These randomized controlled trials were conducted with a small number of patients at a single center, 1 highly experienced endoscopist, and no independent judgment of hemostasis in both groups. Conversely, our study was a multicenter (7 facilities) trial. Our results may be universally applicable because we set a sufficient sample size and ESD was performed by multiple endoscopists. Notably, this study included a third-party evaluation committee that objectively evaluated the data. In a previous study (20), the hemostatic effects of TDM-621 were compared with those of an ESD knife. In this study, because bleeding from an unknown site was targeted, the hemostatic effects of TDM-621 were more accurately compared with those of hemostatic forceps. Furthermore, almost all procedures were classified because “TDM-621 could be applied appropriately to the target site”; thus, operability was easy.

The incidence of AE was not significantly different between the TDM-621 and control groups. No defects derived from TDM-621 treatment were observed. No AE requiring discontinuation of the clinical trial or leading to death occurred. These findings confirm the safety of using TDM-621 for the treatment of blood oozing occurring during ESD, which had been established in previous clinical trials (18–21). Furthermore, stomach ulcers induced by ESD are expected to heal within 1 week of the procedure in 96% of patients and reach the scarring stage in 98% of patients within 8 weeks (22).

This study has several limitations. First, the trial was not double-blind because the purpose of this study was to compare the hemostatic methods. Second, the study targeted only blood oozing and did not include blood spurting. It may be difficult for TDM-621 to completely stop the latter. However, based on our experience, we believe a coagulation hemostat would stop blood spurting because the bleeding site would be easily identified. In addition, it may be possible to reduce blood spurting with TDM-621 (23). Regarding blood spurting, further studies are needed.

In conclusion, TDM-621 was shown to be a useful hemostatic peptide with easy operability and no crucial technical and safety problems in the endoscopic treatment of gastric and rectal tumors. We expect that TDM-621 alone will be applied to a wide range of clinical fields as a hemostatic agent in the future.

## CONFLICTS OF INTEREST

**Guarantor of the article:** Toshio Uraoka, MD, PhD.

**Specific author contributions:** T.U.: conception and design, collection, interpretation, and analysis of the data, and drafting of the article. N.U., T.O., Y.S., N.Y., A.F., and Y.K.: collection and interpretation of the data and drafting of the article. K.M., T.H., and Y. Y.: a third-party evaluation committee. All authors reviewed, contributed to, and approved the final version of the manuscript.

**Financial support:** The work was supported by 3-D Matrix.

**Potential competing interests:** 3-D Matrix contracted and paid all hospitals on the basis of good clinical practice. T.U. and H.T. received consulting fees from 3-D Matrix. Y.N. received consulting fees from Olympus, Boston Scientific, Top, and Fuji film. Y.N. is a patent coholder with Olympus, Pentax, and Top. Y.N. reviewed a research grant from Kaigen Pharmaceutical. T.U and H.T. received lecture fees from Olympus Medical and Fujifilm Medical. K.M. received lecture fees from Olympus. N.U. received lecture fees from Olympus, Fujifilm, Boston Scientific Japan, Daiichi-Sankyo, Takeda Pharmaceutical, EA Pharma, Otsuka Pharmaceutical, AstraZeneca, and Miyarisan Pharmaceutical. Y.N. received lecture fees from Olympus, EA Pharma, Takeda Pharmaceuticals, Ostuka Pharmaceuticals, Astra Zeneca, and Daiich-Sankyo. The remaining authors have no relevant conflicts or financial relationships to report.

**Ethics:** This study was conducted with the approval of the Institutional Review Board of each facility in compliance with relevant laws and regulations, including the Declaration of Helsinki and the Ministerial Ordinance on Good Clinical Practice for Medical Devices. Before conducting the study, the principal investigator or subinvestigators explained the details of the study to the patients and obtained their written informed consent.

**Clinical trial registry:** The study was registered in the UMIN clinical trial registration system (Unique ID issued by UMIN: UMIN000042009).

## Supplementary Material

## Notes

Study Highlights WHAT IS KNOWN

✓ Intraoperative bleeding occurs commonly with tumor resection, especially during rectal and gastric endoscopic submucosal dissection (ESD).
✓ Management of intraoperative bleeding with hemostatic forceps is crucial to the success of ESD.
✓ TDM-621, a synthetic, self-assembling peptide derived from nonliving sources, may help with hemostasis.

WHAT IS NEW HERE

✓ The number of coagulations with hemostatic forceps was significantly reduced in those who received TDM-621 in this trial.
✓ Hemostasis was achieved with TDM-621 alone in 62% of patients.
